# Semiallogenic fusions of MSI^+ ^tumor cells and activated B cells induce MSI-specific T cell responses

**DOI:** 10.1186/1471-2407-11-410

**Published:** 2011-09-26

**Authors:** Yvette Garbe, Ulrike Klier, Michael Linnebacher

**Affiliations:** 1 Institute of Applied Tumor Biology, Ruprecht-Karls University, Heidelberg, Germany; 2 OncoRay - National Center of Radiation Research in Oncology, Medical Faculty Carl Gustav Carus, TU Dresden, Germany; 3 Molecular Oncology and Immunotherapy, Department of General Surgery, University of Rostock, Rostock, Germany

**Keywords:** Cell fusion, frameshift antigens, microsatellite instability, T cell epitopes

## Abstract

**Background:**

Various strategies have been developed to transfer tumor-specific antigens into antigen presenting cells in order to induce cytotoxic T cell responses against tumor cells. One approach uses cellular vaccines based on fusions of autologous antigen presenting cells and allogeneic tumor cells. The fusion cells combine antigenicity of the tumor cell with optimal immunostimulatory capacity of the antigen presenting cells.

Microsatellite instability caused by mutational inactivation of DNA mismatch repair genes results in translational frameshifts when affecting coding regions. It has been shown by us and others that these mutant proteins lead to the presentation of immunogenic frameshift peptides that are - in principle - recognized by a multiplicity of effector T cells.

**Methods:**

We chose microsatellite instability-induced frameshift antigens as ideal to test for induction of tumor specific T cell responses by semiallogenic fusions of microsatellite instable carcinoma cells with CD40-activated B cells. Two fusion clones of HCT116 with activated B cells were selected for stimulation of T cells autologous to the B cell fusion partner. Outgrowing T cells were phenotyped and tested in functional assays.

**Results:**

The fusion clones expressed frameshift antigens as well as high amounts of MHC and costimulatory molecules. Autologous T cells stimulated with these fusions were predominantly CD4^+^, activated, and reacted specifically against the fusion clones and also against the tumor cell fusion partner. Interestingly, a response toward 6 frameshift-derived peptides (of 14 tested) could be observed.

**Conclusion:**

Cellular fusions of MSI^+ ^carcinoma cells and activated B cells combine the antigen-presenting capacity of the B cell with the antigenic repertoire of the carcinoma cell. They present frameshift-derived peptides and can induce specific and fully functional T cells recognizing not only fusion cells but also the carcinoma cells. These hybrid cells may have great potential for cellular immunotherapy and this approach should be further analyzed in preclinical as well as clinical trials. Moreover, this is the first report on the induction of frameshift-specific T cell responses without the use of synthetic peptides.

## Background

The last decades have witnessed the identification of an increasing number of truly specific tumor antigens. Not all antigens carried by human neoplasias have similar immunogenic properties. Somatic mutations should have the highest immunological impact. Such mutations create neoantigenic epitopes which are completely foreign to the immune system and can serve as antigenic determinants. The presence of high-grade microsatellite instability (MSI^+^), for instance, is evidence of ongoing mutagenesis in a fraction of colorectal cancer (CRC). MSI occurs subsequent to DNA mismatch repair inactivation and causes insertion or deletion mutations at short repetitive DNA sequences located throughout the genome. MSI^+ ^tumors are typically infiltrated by predominantly activated cytotoxic T lymphocytes and display increased neoplastic cell apoptosis. These features argue for a strong antitumoral immune response directed against potent tumor rejection antigens [1-3]. We and others demonstrated that frameshift-neopeptides (FSP) encoded by mutations of microsatellites located in coding sequences are highly immunogenic [4-10]. These studies documented that FSPs represent true MSI^+ ^tumor-specific antigens.

Clinical cancer vaccination studies are essentially based on the knowledge of at least one tumor specific antigen. However, reported response rates from those trials are unsatisfying. Among the reasons made responsible for failures are immune evasion of tumor cells, disease-specific immune suppression and poor intrinsic immunogenicity of many tumors.

Cellular fusions of antigen-presenting cells (APC) with tumor cells are a relatively simple and effective way to obtain highly immunogenic vaccines which combine the antigen-presenting properties of professional APC with a full repertoire of tumor antigens [11-14]. Proof-of-principle clinical studies have also been performed [15-17].

Most researchers have focused on dendritic cells as APCs. However, antigen-unspecific B cells can be used as an alternative source of efficient APCs when properly activated by engagement of CD40 [18,19]. Arguments in favour of these CD40-activated B cells (CD40 Bs) are ease of isolation, activation and expansion [20].

Very recently, we optimized the generation of cellular fusions consisting of CD40 Bs and MSI^+ ^CRC cells [21]. In the present study, we have evaluated the potency of T cell induction by semiallogenic cell fusions of a MSI^+ ^tumor cell line and CD40 Bs. In particular, we have examined the potency of *in vitro *induction of T cells specifically recognizing MSI-induced FSPs derived from the tumor cell line fusion partner. The data presented here show that MSI^+^/CD40 B cell hybrid cells induce potent anti-MSI T cell responses, indicating a great potential as immunogens in cancer immunotherapy and providing a rationale for future use in clinical trials.

## Methods

### Cell lines and peptides

All tumor cell lines were obtained from ATCC and from cell line services (Eppelheim, Germany) and grown in RPMI 1640 medium supplemented with 10% fetal calf serum, 2 mmol/L L-glutamine and antibiotics. Tissue culture media and supplements were purchased from PAA (Cölbe, Germany) unless indicated otherwise.

Peptides were obtained from the Peptide Synthesis Facility at the DKFZ. They were dissolved in DMSO and further diluted in PBS. Peptides spanned the entire frameshift-sequence of Caspase-5 (-1) (40 mer; containing 25 aa neo- and 15 aa wt-sequence), OGT (-1) (34 mer; 19 aa neo-, 15 aa wt), Sec63 (-1) (42 mer; 27 aa neo-. 15 aa wt), TGFβRII (-1) (49 mer; 34 aa neo-, 15 aa wt), Taf1B (-1) (40 mer; 25 aa neo-, 15 aa wt), HT001 (-1) ( 47 mer; 32 aa neo-, 15 aa wt), MSH-3 (-1) (40 mer; 31 aa neo-, 9 aa wt) and AIM2 (-1) (20 mer; 13 aa neo-, 7 aa wt). Overlapping 20 mer peptides were synthesized for U79260 (-1) (49 aa neo-, 1 aa wt); AC-1 (-1) (58 aa neo-, 2 aa wt); FLJ11053 (-1) (32 aa neo-, 9 aa wt) together with FLJ378 (-2) (37 aa neo-, 9 aa wt); DAMS (-1) (14 aa neo-, 9 aa wt) together with DAMS (-2) (30 aa neo-, 9 aa wt) and UST3 (-1) (66 aa neo-, 4 aa wt) respectively (for peptide sequences see [9]).

### B cell and T cell purification

Peripheral blood mononuclear cells were isolated from heparinized blood of a healthy HLA-A02^+ ^donor using Ficoll-density gradient centrifugation. Whole CD3^+ ^T cells were obtained from PBMCs by magnetic depletion of non-T cells using the MACS Pan T Cell Isolation Kit II (Miltenyi; Bergisch Gladbach, Germany) according to manufacturer's instructions. The remaining non-T cells were subsequently used as a source for B cells. All procedures using human cells were approved by the Ethics Committee of the University of Heidelberg in accordance with the provisions of the declaration of Helsinki (as revised in Edinburgh 2000). An informed consent in written was obtained prior to the blood sampling procedure.

### Culture of CD40 Bs

The culture of CD40 Bs was performed as described [22]. Briefly, B cells were stimulated via NIH3T3 stimulator cells stably expressing human CD154. Lethally irradiated stimulator cells (60 Gy) were plated on 6-well plates (0.35 × 10^5 ^cells/well) and cultured overnight. After removing medium, magnetic depleted non Tc (2 × 10^6 ^cells/ml) were resuspended in Iscove's modified DMEM (IMDM) supplemented with 10% human AB serum, 5 μg/ml insulin, 50 μg/ml transferrin and 15 μg/ml gentamicin in the presence of IL-4 (10 IU/ml) and cyclosporin A (5.5 × 10^-7^M). Every 3-5 days cells were transferred to new plates containing fresh irradiated stimulator cells. All cytokines were obtained from PromoCell, Heidelberg, Germany.

### Fusion of MSI^+ ^tumor cells with CD40 Bs

HCT116 tumor cells and CD40 Bs from a healthy HLA-A02^+ ^donor were washed in serum-free RPMI 1640 medium, pelleted and stained with 5 μM 5-chloromethylfluorescein diacetate or 5 μM 5-(and 6)-(((4-chloromethyl) benzoyl)-amino) tetramethyl-rhodamin in PBS, respectively. Cells were incubated for 45 min at 37°C, washed and resuspended in serum-free RPMI 1640 medium. After a second incubation period, stained cells were resuspended in serum-free RPMI 1640. CD40 Bs were mixed with tumor cells at a CD40 Bs:tumor cell ratio of 2:1 and exposed 5 min to 4 × 10^-5^M SDS. Cell suspension was pelleted and 1 ml 50% polyethylene glycol 1500 (Roche, Mannheim, Germany) was added over 5 min to the CD40 Bs/tumor cell pellet while stirring the cells continuously. The polyethylene glycol solution was then diluted by slow addition of first 1 ml warmed serum- and phenol red-free medium over 3 min with continued stirring and then another 10 ml of this medium over 5 min. After centrifugation cells were resuspended in phenol red-free RPMI 1640 supplemented with 10% FCS. Cells were cultured 24 h before the fusion efficacy was analyzed by flow cytometry. Fused cells consisting of MSI^+ ^colon carcinoma cells HCT116 and CD40 Bs, which exhibited dual fluorescence, were cloned by limiting dilution. Briefly, fused cells were seeded under limiting dilution conditions (0.7 cells/well) in 96-well plates containing a final volume of 200 μl RPMI 1640 with 10% FCS. Outgrowing fusion clones were comprehensively screened concerning the cell surface expression of HLA-A02, MHC class I and II molecules and costimulators (CD40, CD80, and CD86). For all subsequent experiments, two clones were selected and designated as Fc1 and Fc2.

### T cell stimulation with semiallogenic cellular fusion clones

Fusion clones were collected from cell culture, irradiated (200 Gy) and added to purified CD3^+ ^autologous T cells at a ratio of 3:1 (Tc:fusion cell) in IMDM supplemented with 10% human AB serum, 5 μg/ml insulin, 50 μg/ml transferrin and 15 μg/ml gentamicin in the presence of IL-7 (10 IU/ml ). Cells were plated at a density of 2 × 10^6 ^T cells in 1 ml of medium per well of a 24 well plate. For T cell restimulation this was repeated weekly. IL-2 was first given at day 21 (10 IU/ml), also at day 24 and from day 28 on only IL-2 was used instead of IL-7.

CD4^+ ^T cells and CD8^+ ^T cells were obtained from the whole T cell population by magnetic cell sorting using MACS CD4 and CD8 microbeads (Miltenyi; Bergisch Gladbach, Germany) according to manufacturer's instructions. CD4 microbeads were used for the sorting of CD8^+ ^T cells and CD8 microbeads for the sorting of CD4^+ ^T cells. Selection of T cells was checked by flow cytometry. CD4^+ ^T cells and CD8^+ ^T cells were restimulated separately with irradiated fusion clones and IL-2 as described for the whole T cell population.

### IFN-γ ELISpot assay

ELISpot assays were performed using nitrocellulose-96-well plates (Multiscreen; Millipore, Bedford, USA) covered with mouse anti-human IFN-γ mAb (Mabtech, Sweden) and blocked with serum containing medium. Initially, *in vitro *primed T cells (1 × 10^4^) were stimulated in triplicates with 2 × 10^4 ^CD40 Bs, HCT116, Fc1 or Fc2 cells per well as targets. Afterwards T effector cells (1 × 10^4^) were plated in sixplicates with 2 × 10^4 ^peptide-loaded autologous CD40 Bs. Peptides were added at a final concentration of 10 μg/ml. After incubation for 16 h at 37°C, plates were washed, incubated with biotinylated rabbit anti-human IFN-γ for 4 h, washed again, incubated with streptavidin-alkaline phosphatase for 2 h, followed by a final wash. Spots were detected by incubation with NBT/BCIP (Sigma-Aldrich, Steinheim, Germany) up to 1 h. Reaction was stopped with water and after drying spots were counted. The deduced frequency of peptide-specific T cells was calculated by subtracting mean numbers of spots in the no-peptide control from mean numbers of spots in the peptide-stimulated sample. Negative values were scored as zero.

After proving the assumption of normality, differences between negative control and FSP-containing wells were determined by using the unpaired Student's t-test. If normality failed, the nonparametric Mann-Whitney U-Test was applied. The tests were performed by using Sigma-Stat 3.0 (Jandel Corp, San Rafael, CA). The criterion for significance was set to p < 0.05.

### Determination of cMS mutation pattern

Genomic DNA was isolated from microdissected tumor sections using the DNeasy Tissue Kit (Qiagen, Hilden, Germany). For analysis of cMS, the corresponding genomic regions were amplified as described previously [23]. PCR products were analyzed using an ABI3100 genetic analyzer and Genescan Analysis Software (Applied Biosystems, Darmstadt, Germany). Instability was scored, if comparison with amplification products of normal tissue revealed the occurrence of novel peaks or if the ratio of corresponding peak areas was ≤0.5 or ≥2.

### Cytotoxicity assays

Standard chromium release assays were performed as described [24]. Tumor target cells were labelled with 100 μCi [^51^Cr]-sodium chromate for one hour at 37°C. For each experimental condition, cells were plated in V-bottomed 96-well plates with 10^3 ^target cells/well in triplicate. Varying numbers of CTL were added to a final volume of 200 μl and incubated for 4 h at 37°C. Spontaneous and maximal release was determined in the presence of medium alone or of 1% NP-40. Supernatants (100 μl/well) were harvested and counted in a gamma-counter. The percentage of specific lysis was calculated as follows: 100% × [experimental release - spontaneous release]/[maximal release - spontaneous release].

### FACS analysis

Expression of the following surface markers was analyzed: MHCI (W6/32), HLA-A2 (BB7.2), MHCII (12G6), CD3 (OKT3), CD28 (CD28.2) with unlabeled primary antibodies and FITC-labelled goat anti-mouse IgG's (Dianova, Hamburg, Germany) as second antibody. Cells treated without primary antibody were used as negative control. CD4 (RPA-T4), CD8 (RPA-T8), CD19 (HIB19), CD45RO (UCHL1), CD50 (101-1D2, Cymbus Biotechnology, UK), CD58 (1C3 AICD58.6), CD80 (BB1/B7-1), CD86 (B70/B7-2) and CD102 (B-T1; Serotec, Oxford, UK) were directly FITC-conjugated, whereas CD25 (M-A251), CD69 (FN50) and CD71 (SOM4D10, Diatec, Oslo, Sweden) were PE-conjugated. Isotype-matched monoclonal antibodies were used as a negative control.

For intracellular staining, T cells were incubated with the protein transport inhibitor brefeldin A (2 μg/ml, Serva, Deisenhofen, Germany) for 15 h at 37°C. After two washes with PBS/1% FCS, cells were fixed with cold 4% paraformaldehyde (PFA, Serva) in PBS for 10 min at 4°C, washed twice with PBS/1% FCS and permeabilized with saponine buffer (PBS, 0,1% saponine, 1% FCS and 0,01 M Hepes) for 10 min at room temperature. T cells were subsequently stained for intracellular cytokines with PE-labelled anti-IFN-γ (4SB3), anti-TNF-α(Mab11), anti-IL-2 (MQ1-17H12) mAbs, or IgG1 isotype control mAb in saponine buffer for 20 min at 4°C. Cells were washed twice with saponine buffer and resuspended in PBS/1% FCS before measurement. Intracellular perforin was detected with PE-labelled anti-perforin (dG9) mAb. For analysis of intracellular granzyme B, cells were incubated with anti-granzyme B (2C5/F5, Serotec) mAb followed by FITC-labelled goat anti-mouse IgG's. Cells treated without primary antibody were used as negative control. Cell surface and intracellular immunofluorescences were obtained using FACSCalibur (BD Biosciences, Heidelberg, Germany) and CellQuest software. Typically, 20.000 cells were acquired from each sample. Cells to be analyzed were first gated according to reasonable size and granularity in the forward/sideward scatter plot. Gated cells were than blotted into histogram blots. Mean intensities of the negative control were between 2 and 10. For determination of percentages of positive cells, the cut-off included a maximum of 2% of the events in the negative control. Antibodies and signal detection reagents were obtained from BD Biosciences unless stated otherwise.

## Results

### Semiallogenic cellular fusion and selection of fusion cell clones

The CD40 Bs used to generate fusions were derived from a healthy volunteer with neither a family history of hereditary non-polyposis colorectal cancer nor any other known severe disease, in particular no tumor. Therefore, the T cells of this donor can be considered as naive with regard to prior contact with MSI-induced frameshift mutations. As tumor cell fusion partner, the MSI^+ ^CRC cell line HCT116 was chosen because the mutational status of many coding microsatellites (cMS) is well known (Table 1), it shares human leukocyte antigen (HLA)-A02 as restriction element with the CD40 Bs of the healthy donor and it has been shown to be accessible to lysis by cytotoxic T lymphocytes (CTL) [8,10]. Semiallogenic cellular fusions were performed in the presence of polyethylene glycol. The fusion efficiencies were between 10 and 15% [data not shown] and thus very similar to published data [21]. However, additionally to fresh fusions we decided to obtain fusion cell clones as this allowed us to select variants expressing mutations in cMS giving rise to FSPs with high immunogenic potential and additionally high amounts of major histocompatibility complex (MHC) and of the costimulatory molecules CD40, CD80 and CD86. Extensive characterization of established fusion clones was performed and two fusion cell clones (Fc1 and Fc2) expressing many FSPs (Table 1) and higher amounts of MHC and costimulatory molecules as HCT116 (Table 2) were selected as stimulator cells in subsequent T cell stimulations. Data of two fusion clones expressing low amounts of MHC and costimulatory molecules are additionally shown (Table 2).

**Table 1:** Analysis of Microsatellite instability (MSI)

Microsatellite markers	HCT116	Fc1	Fc2
**BAT25 **^**1**^	+	+	+
**BAT26 **^**1**^	+	+	+
**MFD15 **^**1**^	+	+	+
**D5S346 **^**1**^	+	-	-
**D2S123 **^**1**^	+	+	-

**CASP5**	wt/-1	wt/-1	wt
**OGT**	wt	wt	wt
**SEC63**	wt	wt/-1	wt/-1
**TGFB2R**	wt	wt	wt
**Taf1B**	-3/-2/wt	-2/wt	-2/wt
**HT001**	-1/-2	-1	-1
**MSH3**	-1	-1	-2
**U79260**	-4	-4	-5
**AC-1 (c4orf6)**	wt	wt	wt
**AIM2**	-1	-1	wt/-1
**FLJ20378**	-3	-3	-3
**FLJ11053**	wt/-1	wt/-1	wt
**DAMS**	n.d.	n.d.	n.d.
**UST3**	-2	wt/-1	wt/-2

^1 ^MSI is indicated by a "+".

^2 ^length of cMS alleles analyzed are indicated.

Wt (wildtype) and observed length variations are displayed.

**Table 2:** Expression of cell surface molecules on CD40 Bs, HCT116 and fusion clones 1 to 4

	mean fluorescence intensity					
**antigen**	**CD40 Bs**	**HCT116**	**Fc1**	**Fc2**	Fc3	Fc4

**HLA-A0201**	1 285.5	74.4	163.9	177.5	575.5	176.7
**MHC class I**	1 768.0	496.9	1 006.0	909.5	1736.8	800.8
**MHC class II**	524.8	7.4	39.1	40.5	11.9	9.0
**CD80**	179.8	6.3	9.7	16.5	4.8	8.5
**CD86**	117.4	5.4	11.7	13.5	5.7	7.7
**CD40**	393.9	21.0	50.2	50.5	44.2	27.8

Fc1 and Fc2 have been used in subsequent experiments

Fc3 and Fc4 have not been analysed further because of low expression of MHC class II.

### In vitro T cell stimulations

**Figure 1:**
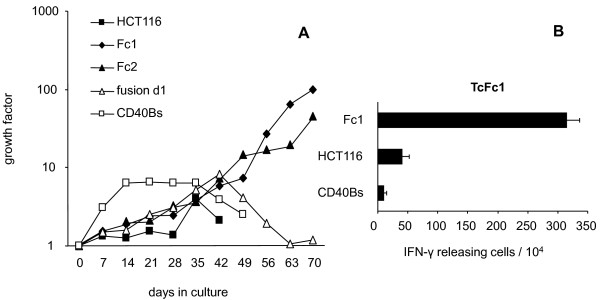
**Characteristics of T cells stimulated with fusion and control cells**. **A**. Growth curve of T cells stimulated with fresh fusions (d1), Fc1, Fc2, autologous CD40 Bs and HCT116 cells. Fusion clones (Fc1 and Fc2) generated from autologous CD40 Bs and HCT116 were able to stimulate long-lasting growth of autologous T cells compared to CD40 Bs or HCT116 alone. Days in culture are given together with the calculated growth factor accumulated over the culture time. **B**. ELISpot analysis of T cells stimulated with Fc1. T cells (1 × 10^4 ^cells/well) were stimulated with 2 × 10^4 ^CD40 Bs, HCT116 and Fc1 cells per well as targets. Analysis was performed in triplicates. The number of IFN-γ releasing activated T cells for the total number of cells analyzed (10^4^) is given together with the standard deviation.

Isolated peripheral T cells autologous to the CD40 Bs used as fusion partner were weekly stimulated with fresh fusions, with Fc1 and Fc2 using an established protocol of *in vitro *T cell stimulation [10]. As controls, T cells were also stimulated with autologous CD40 Bs and with HCT116 cells. In the first five weeks, all bulk T cell cultures grew moderately but in the following two weeks the T cells stimulated with HCT116 and CD40 Bs decreased followed by the fresh fusions stimulated T cells (Figure 1A). Sustained growth with fold increases exceeding 10 was reached solely by the T cell cultures stimulated with the fusion cell clones Fc1 and Fc2 (Figure 1A). Consequently, all subsequent detailed analyses were restricted to the latter two cultures. Phenotypical analysis proved that the cultures consisted of pure T cells with a slightly dominance of CD4^+ ^cells but a clearly activated status judged from the high expression levels of CD25, CD45RO, CD69 and CD71 (Table 3). The Fc1-stimulated T cells produced higher levels of interferon (IFN)-γ and Perforin. Granzyme B was detectable in high levels in both T cell populations (Table 3).

**Table 3:** FACS analysis of Tc generated against Fc1 and Fc2

	% positive cells	
**antigen**	**TcFc1**	**TcFc2**

**CD3 **^**1**^	97.0	97.5
**CD4 **^**1**^	53.9	57.4
**CD8 **^**1**^	46.0	43.2
**CD28 **^**1**^	37.2	43.6
**CD45RO **^**1**^	79.7	81.7
**CD25 **^**1**^	27.5	28.2
**CD69 **^**1**^	29.3	36.1
**CD71 **^1^	89.2	89.2
**IFN-γ **^**2**^	26.9	15.3
**Perforin **^**2**^	25.8	3.9
**Granzyme B **^2^	63.7	74.4

^1 ^d37 of Tc culture

^2 ^d72 of Tc culture

### Fc1 and Fc2 T cell cultures recognize tumor target cells

**Figure 2:**
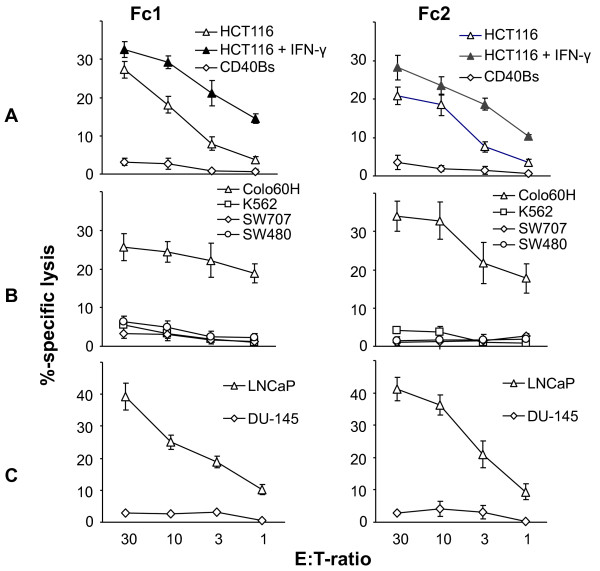
**Cytotoxic reactivity of Fc-stimulated T cells**. T cells were generated against Fc1 (TcFc1, left) and against Fc2 (TcFc2, right). **A**. Specific cytotoxic activity against the MSI^+^, HLA-A02^+ ^colon carcinoma cell line HCT116 is shown with and without prior IFN-γ-treatment (200 U/ml). Both TcFc1 and TcFc2 cultures lack reactivity towards autologous CD40 Bs. **B**. Specific lysis of MSI^+^, HLA-A02^+ ^colon carcinoma cell line Colo60H is shown in contrast to that of microsatellite stable, HLA-A02^+ ^colon carcinoma cell lines SW707 and SW480. Lack of reactivity against NK sensitive K562 excludes NK cell activity. **C**. Cytotoxic potential of TcFc1 and TcFc2 towards the MSI^+^, HLA-A02^+ ^prostate carcinoma cell line LNCaP compared to the MSI^+ ^HLA-A02^- ^prostate carcinoma cell line DU-145. Target cells were labelled with [^51^Cr]-sodium chromate for 1 h. Different effector to target cell ratios (E:T ratio) are shown. All results are displayed as the mean and standard deviation from three replicate wells.

Next, we addressed whether the T cells recognized the fusion clones used for stimulation. A representative ELISpot-analysis of Fc1-stimulated T cells is presented in Figure 1B. As expected, the T cells readily secreted IFN-γ in response to Fc1 (3.2%). The autologous B cells were not recognized (< 0.1%) but HCT116 could provoke a minor reaction (0.4%). This weak reaction towards the tumor cell fusion partner HCT116 did however not prevent killing of HCT116 by Fc1 and Fc2-stimulated T cells as subsequently tested in cytotoxicity experiments (Figure 2A). Of note, this killing could be enhanced by pre-treatment of HCT116 with IFN-γ leading to an upregulation of MHC molecules and immune presentation (Figure 2A). This activity was unlikely due to contaminating NK cells in the T cell bulk cultures, since the classical NK cell target cell line K562 was not recognized (Figure 2B). Testing of another three HLA-A2^+ ^colorectal cell lines revealed no recognition of the MSI^- ^cell lines SW480 and SW707 but recognition of the MSI^+ ^cell line Colo60H (Figure 2B). Finally, Fc1 and Fc2-stimulated T cells' reactivity was tested against the MSI^+ ^prostate carcinoma cell lines LNCaP (HLA-A2^+^) and DU-145 (HLA-A2^-^) (Figure 2C). Both T cell lines strongly reacted against the MSI^+ ^and HLA-A2^+ ^cell line LNCaP whereas they did not kill the MSI^+ ^but HLA-A2^- ^DU-145 (Figure 2C).

### Fc1CD4^+ ^T cells have higher proliferative and Fc1CD8^+ ^T cells higher cytotoxic potential

**Figure 3:**
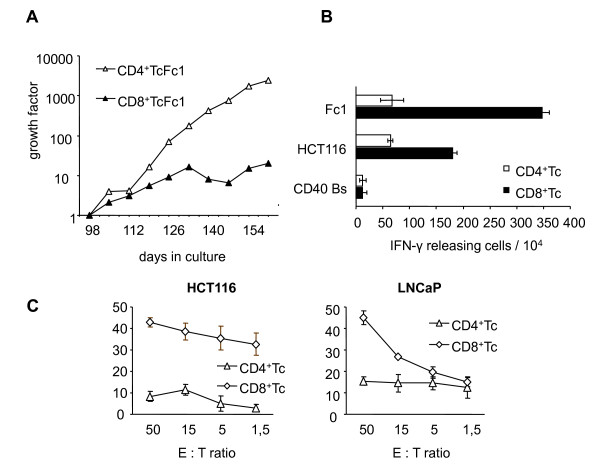
**Analysis of selected CD4**^**+ **^**and CD8**^**+ **^**Fc-stimulated T cells**. **A**. Growth of CD4^+ ^and CD8^+ ^T cells stimulated with Fc1 after magnetic separation. Total days in culture are given together with the calculated growth factor accumulated over the culture time starting after magnetic separation. **B**. ELISpot analysis of CD4^+ ^and CD8^+ ^T cells generated against CD40 Bs/HCT116 fusion clone 1 (Fc1). T cells (1 × 10^4 ^cells/well) were stimulated with 2 × 10^4 ^CD40 Bs, HCT116 or Fc1 as targets. The number of specific IFN-γ releasing activated T cells (spots) among the total number of T cells analyzed (10^4^) and the standard deviation from three replicate wells are given. **C**. Cytotoxic activity of CD4^+ ^and CD8^+ ^T cells (CD4^+ ^Tc, CD8^+ ^Tc) generated against CD40 Bs/HCT116 fusion clone 1. **Left**: Specific reactivity of CD4^+ ^Tc and CD8^+ ^Tc against MSI^+ ^HLA-A02^+ ^colon carcinoma cell line HCT116. **Right**: Specific lytic potential of CD4^+ ^Tc and CD8^+ ^Tc against the MSI^+ ^HLA-A02^+ ^prostate carcinoma cell line LNCaP. Targets were labelled with [^51^Cr]-sodium chromate for 1 h. Different effector to target cell ratios (E:T ratio) are shown. All results are displayed as the mean and standard deviation from three replicate wells.

**Figure 4:**
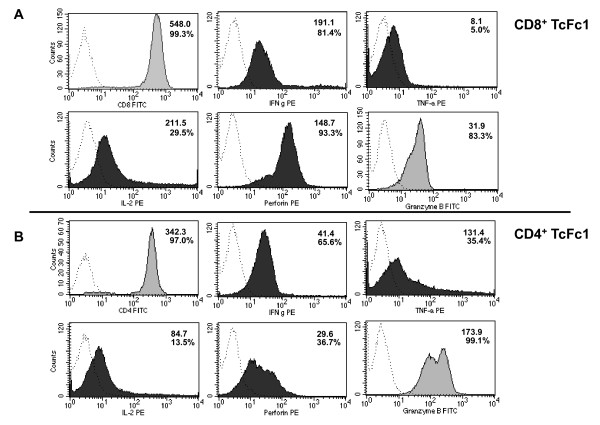
**FACS analysis of CD4**^**+ **^**and CD8**^**+ **^**Fc-stimulated T cells**. After magnetic separation of CD4 and CD8 T cells followed by two additional rounds of stimulation with Fc1, the T cells were analyzed for expression of CD4/CD8 and of the indicated intracellular cytokines. A. Fc1 CD8^+ ^T cells B. Fc1 CD4^+ ^T cells. The percentages of positive cells are indicated together with the mean fluorescence intensities. Isotype matched negative controls are indicated by dotted lines.

In order to elucidate the contribution of CD4^+ ^and CD8^+ ^T cells towards tumor cell recognition, we negatively separated the Fc1-stimulated T cells magnetically to high purity. As can be depicted from Figure 3A, the CD4^+ ^T cells Fc1 had greater proliferative potential. However, the CD8^+ ^T cells Fc1 better recognized HCT116 as well as LNCaP cells both in IFN-γ ELISpot as well as in cytotoxicity experiments (Figures 3B and 3C). As an additional observation, the better recognition of Fc1 compared to HCT116 could be attributed to the CD8^+ ^T cells Fc1 (Figure 3B). These functional data match with the results of phenotypic FACS analysis. CD8^+ ^TcFc1 showed stronger expression of IFN-γ, interleukin (IL)-2 and perforin (Figure 4A), whereas CD4^+ ^TcFc1 produced more tumor necrosis factor (TNF)-α and granzyme B (Figure 4B).

### Fc1 and Fc2 T cell cultures contain FSP-specific T cells

**Figure 5:**
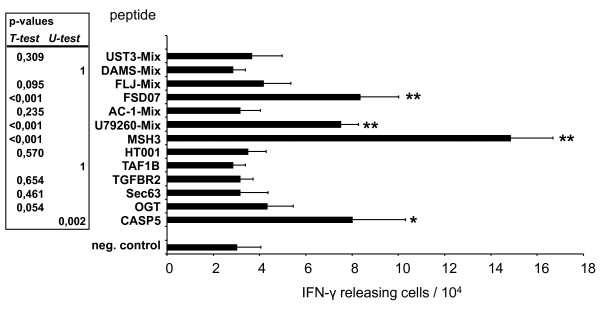
**ELISpot analysis of FSP-recognition of T cells stimulated with Fc1**. T cells (1 × 10^4 ^cells/well) were stimulated with 2 × 10^4 ^peptide loaded autologous CD40 Bs. Peptides were added at a final concentration of 10 μg/ml. Analysis was performed in sixplicates. The mean number of IFN-γ releasing T cells is given together with the standard deviation. Significant reactions in comparison to the negative control are indicated by *.

Since we hypothesized that at least a proportion of the fusion clone stimulated T cells are specific for MSI-induced FSPs, we tested the Fc1-stimulated T cells for recognition of FSPs in IFN-γ ELISpot-assays. Here, we observed a response towards four of the 14 FSPs included into this analysis (Figure 5). This proves that fusions of MSI^+ ^cells with APC can functionally present FSPs to T cells which in turn can be activated and gain the potential to attack MSI^+ ^tumor cells.

## Discussion

Data presented here confirm our previous findings that cell hybrids of human MSI^+ ^tumor cells and CD40 Bs as APC can easily be generated [21]. Fusion cells retain desired characteristics of both fusion partners; in particular the expression of MSI-induced FSPs and of functional antigen processing and presentation machinery together with potent immunostimulatory capacity. In the present study we worked with stable fusion cell clones instead of highly purified fresh fusion cells we described recently [21]. This approach was chosen to ensure a uniform fusion cell population concerning the immunostimulatory phenotype and the expression of FSPs. Indeed, the distribution of these features was heterogeneous between different clones. Others have shown that careful selection of fusion clones may be essential for successful T cell stimulation [25].

Good arguments in favour of CD40 Bs as APC are the possibilities to easily isolate them even out of minimal amounts of peripheral blood, to propagate them for longer time periods and lastly the outstanding T cell stimulatory capacity of fully activated B cells [8,21,22].

The disclosure of the molecular pathways leading to MSI has led us and others to hypothesize and prove that when affecting coding regions, this will ultimately give rise to FSPs with high immunogenic potential [4-10]. Beyond the documentation that FSPs represent true MSI^+ ^tumor-specific antigens, these studies suggest a tumor-immunological model character of MSI^+ ^tumors. Large numbers of specific FSPs have been identified [see [23] for an overview] and many of those are best suited as immunological read out target structures. To the best of our knowledge, such a high number of specific antigens have not been identified for any other tumor entity.

Several studies have shown that fusion cells have the potential to induce CD4^+ ^as well as CD8^+ ^T-cell mediated antitumor immunity, protection from an otherwise lethal challenge of tumor, and even regression of established metastatic disease [15,26-28]. *In vitro *stimulation of CD3^+ ^T cells with the two fusion clones that were analyzed in detail here induced effector T cells with strong cytotoxic potential that is HLA-A02-restricted and mediated mainly by CD8^+ ^T cells. Moreover, these polyclonal T cell responses are directed against MSI^+ ^tumor cells. ELISpot assays revealed that at least a part of this reactivity was due to specific recognition of FSPs. In addition to the four FSPs specifically recognized in the ELISpot analysis shown in Figure 5, we observed significant reactions against two additional FSPs (OGT(-1) and AC-1(-1)) in repetition analysis (data not shown). Lack of reactivity towards microsatellite stable tumor cells but target cell recognition of yet another tumor entity displaying the MSI phenotype strongly argue for MSI^+ ^tumor antigens shared between those tumor entities. Thus, this is the first report on the induction of a specific immune response towards MSI-induced FSPs without the use of artificial peptides.

Another interesting aspect was the comparably stronger reactivity of fusion cell stimulated T cells to the fusion clone than to the HCT116 fusion partner. This could mainly be attributed to the CD8^+ ^T cells. As explanation, we consider a better presentation to the T cells of FSPs by the fusion clones than by the HCT116 tumor cells or a better activation of low avidity T cells as likely.

In conclusion, our findings further support the idea that APC/tumor cell fusions represent an attractive approach to cancer immunotherapy [29,30]. We suggest that semiallogenic cell hybrids of human MSI^+ ^tumor cells and CD40 Bs are a feasible approach to generate polyepitope vaccines with the capacity to induce polyvalent immune responses. Future studies with primary tumor cells of MSI^+ ^patients fused to autologous APCs should further address the feasibility of this approach in a complete autologous situation as this may have important clinical implications for the treatment of MSI^+ ^tumors. However, the *in vivo *efficacy of such cell-based MSI^+ ^cancer vaccines must be proven either in appropriate animal models or in carefully designed small clinical studies including cases of very advanced disease.

## Conclusions

In the presented study we demonstrate the potential of semiallogeneic cellular fusions of MSI^+ ^colorectal carcinoma cells and activated B cells to induce FSP-specific T cells. Selection of fusion cell clones facilitates detailed analysis of the antigen-presenting capacity as well as the repertoire of expressed frameshift antigens. These fusion clones were able to stimulate T cells which showed specificity for the fusion cells themselves and additionally for the MSI^+ ^carcinoma cell fusion partner. Moreover, we were able to demonstrate that at least part of this antitumoral potential was due to the specific induction of T cells recognizing several FSPs. Thus, these data underline the high potential of cellular fusions to induce tumor-specific T cells and may contribute to the development of novel cellular immunotherapies.

## List of abbreviations

APC: antigen presenting cell; CD40 Bs: CD40-activated B cells; cMS: coding microsatellite; CRC: colorectal cancer; CTL: cytotoxic T lymphocyte; E:T: Effector:target cell ratio; ELISpot: enzyme linked immunospot; Fc: fusion cell clone; FSP: frameshift-neopeptide; HLA-A2: human leukocyte antigen A2; IFN: interferon; IL: interleukin; MHC: major histocompatibility complex; MSI^+^: microsatellite instability positive; TNF: tumor necrosis factor

## Competing interests

The authors declare that they have no competing interests.

## Authors' contributions

YG carried out most of the experiments, participated in data analysis and manuscript drafting. UK participated in data analysis and helped to draft the manuscript. ML designed and coordinated the study and helped in selected experiments and to draft the manuscript. All authors read and approved the final manuscript.

## Pre-publication history

The pre-publication history for this paper can be accessed here:

http://www.biomedcentral.com/1471-2407/11/410/prepub
